# The Association between Gut Microbiota and Its Metabolites in Gestational Diabetes Mellitus

**DOI:** 10.4014/jmb.2403.03064

**Published:** 2024-07-24

**Authors:** Hua Lin, Changxi Liao, Rujing Zhang

**Affiliations:** 1 Department of Clinical Laboratory, the Affiliated Hospital of Putian University, Putian, Fujian 351100, P.R. China; 2 Key Laboratory of Medical Microecology (Putian University), Fujian Province University, Putian, Fujian 351100, P.R. China; 3 Department of Hepatobiliary Surgery, the Affiliated Hospital of Putian University, Putian, Fujian 351100, P.R. China

**Keywords:** Gestational diabetes mellitus, gut microbiome, fecal metabolomics, phenylalanine metabolism, nucleotide metabolism

## Abstract

Gut microbial metabolites have been demonstrated to play a role in diabetes mellitus and gestational diabetes mellitus (GDM). This study aimed to investigate gut microbiome, fecal metabolomics, and their association in pregnant women with and without GDM. The metabolome indicated that the top 2 differential metabolic pathways between control (Con) and GDM groups were phenylalanine metabolism and nucleotide metabolism. The increased Phenylalanylglycine, m-coumaric acid, and Phenylacetic acid were among the top differential metabolites between Con and GDM groups and involved in phenylalanine metabolism. Uracil and hypoxanthine were top differential metabolites in Con vs. GDM and involved in nucleotide metabolism. The proficiently altered gut microbiota at the class level was c_unclassified_ Firmicutes. Association analysis between gut microbiota and fecal metabolites indicated that the increased gut symbiont Clostridium belonged to Firmicutes and was linked to the dysregulation of phenylalanine metabolism in GDM. This study may provide the mechanism underlying how Clostridium–phenylalanine metabolism association contributes to GDM pathogenesis and also be a novel therapeutic strategy to treat GDM.

## Introduction

Gestational diabetes mellitus (GDM) was referred to as a transient glucose intolerance and hyperglycemia during pregnancy and often occurred at 24–28 weeks of gestation. GDM affects around 16.5% of pregnant women worldwide, and this number is expected to increase as the obesity epidemic continues to escalate [1]. Without intensive intervention, GDM would increase the risk of type 2 diabetes and maternal cardiovascular disease, and infant birth complications. Even with a successful birth, children also have long-term risks of obesity, cardiovascular disease and type 2 diabetes, etc. Therefore, GDM is a heavy health burden for both mothers and children [2]. One of the mechanisms underlying GDM is an imbalance between inadequate insulin secretion and the placental secretion of diabetogenic hormones, which decreases insulin sensitivity during pregnancy[3].

Increasing evidence indicated that gut microbiota influences GDM and other metabolic diseases including obesity and type 2 diabetes mellitus(T2M) [4]. The gut microbiota, a dynamic and complex population of microorganisms, harbors in the human gastrointestinal (GI) tract. It exerts a marked effect on the host during homeostasis and disease [5]. Research suggested that the gut microbiota regulated glucose metabolism and the dysbiosis of the gut microbiota mediated the T2M progression through the development of insulin resistance, inflammation, and other metabolic disturbances. Furthermore, it was found that the gut microbial sordidly influenced the gut and other organs, and affected the disease pathogenesis [6, 7] and inflammatory responses [8]. Altered gut microbial metabolites including bile acids (BAs), short-chain fatty acids (SCFAs), aromatic amino acids, etc. were involved in the pathogenesis of T2DM [9]. The metabolites can enter the systemic circulation and further regulate multiple metabolic pathways [5, 10]. GDM shares some pathophysiological features with T2M and the dysbiosis of gut microbiota also plays a role in the pathogenesis of GDM [8]. Amounting evidence indicated that dysbiosis could affect insulin resistance, inflammation, and other metabolic disturbances in GDM [3]. However, the mechanistic links between the altered gut microbiome and microbial metabolites to GDM progression are not fully understood. The integrated analyses of microbial metabolites and gut microbiome, and their association linking to host phenotype may provide insight into the development of GDM.

In the present study, we conducted metabolome–microbiome dual-omics analyses in a cohort of pregnant women with normal glucose tolerance to explore the relationship between gut microbiota and its metabolites and the potential mechanism involved in GDM progression.

## Methods

### Subjects and Sample Collection

30 pregnant women with GDM and 30 pregnant women with normal glucose levels were recruited from The Affiliated Hospital of Putian University from August 2021 to January 2023. The diagnosis of GDM was referred to the 75 g oral glucose tolerance test (OGTT) at 24–28th gestational weeks with the criterion [11]: fasting blood glucose (FBG) ≥5.1 mmol/l or 1 h OGTT glucose values ≥10.0 mmol/l or 2 h OGTT glucose values ≥8.5 mmol/l. Subjects with pre-pregnancy diabetes, metabolic diseases, antibiotic use, alcohol abuse or drug abuse within three months, and chronic diseases requiring medication were excluded. Pregnant women with normal glucose levels with matched age, BMI, lifestyle habits, and medical history were designated as the control group. The fecal specimens of 60 subjects were collected after fasting overnight. At least 1-2 g (soybean size) fecal samples were collected in a 2 ml EP tube to minimize sample contamination and DNA degradation, temporarily stored at 4°C, and frozen in a -80°C freezer within 1 hour. This study was approved [No: PUYIFULUN (202416)] by the Human Research Ethics Committee in The First Affiliated Hospital of Putian University and carried out in accordance with the Helsinki Declaration. All participants were informed and signed a written consent before any procedure was performed.

### Metabolite Profile Analysis and Data Processing

The metabolomic processing was performed by NOVOGENE (Tianjin, China, NovoGene.com). The samples were placed in a 4°C automatic sampler during the entire process of analysis. The samples were analyzed with the SHIMADZU-LC30 ultra-high performance liquid chromatography system (UHPLC) and ACQUITY UPLC HSS T3 chromatography column (2.1 × 100 mm, 1.8 μm; Waters, USA). The injection volume: 4 μl; Column temperature: 40°C; flow rate: 0.3 ml/min; Chromatographic mobile phase A: 0.1% formic acid aqueous solution; B: acetonitrile. Each sample was detected in positive (+) and negative (-) ion modes by electric spray ionization (ESI). After UPLC separation, the sample was analyzed by a Thermo Scientific mass spectrometer (QE Plus) and ionized using a HESI source. The raw data was aligned, retention time was corrected, and peak area was extracted with MSDIAL software. Metabolite structure identification was performed using precise mass number matching (mass tolerance <10 ppm) and secondary spectrum matching (mass tolerance < 0.01 Da) with public databases such as HMDB, MassBank, GNPS, and self-built metabolite standard libraries (BP-DB). Normalize the total peak area of the positive and negative ion data separately, integrate the positive and negative ion peaks, and apply Python software for pattern recognition. The data is preprocessed by Unit variance scaling (UV) and then subjected to subsequent data analysis.

The modified MetaboAnalystR 2.0 R package was employed for multivariate and univariate statistical analysis of the metabolites. Multivariate statistical analysis including principal component analysis (PCA) and a supervised orthogonal Partial Least Squares Discrimination Analysis (OPLS-DA) were applied to detect the differences in metabolites between the groups. The R2X, R2Y, and Q2 values were employed for validating the model. The overall variable in the OPLS-DA model was ranked with VIP (variable importance in the projection). Differential metabolites were defined with the standard of VIP > 1, *p* < 0.05, and Fold change (FC) > 1.5. The enrichment pathway analysis for differential metabolites was conducted with the Kyoto Encyclopedia of Genes and Genomes database by the software MetaboAnalystR.

### Gut Microbiome Analysis

Fecal DNA was extracted from fecal samples with QIAamp PowerFecal Pro DNA Kit (Cat: 51804, QIAGEN, USA), and DNA was diluted to 1 ng/μl solution and amplified the 16S rRNA V3–V4 regions using 338F (5'-ACTCCTACGGGAGGCAGCAG-3') and 806 R (5'-GGACTACHVGG-GTWTCTAAT-3') primers [12] with TransStart FastPfu DNA Polymerase (Transgen Biotech, China). We then purified the PCR products using VAHTSTM DNA Clean Beads (Vazyme Inc., China). Next, we applied a TruSeq Nano DNA LT Library Prep Kit (Illumina, USA) to construct the libraries and conducted on the MiSeq platform (Illumina) for library quality assessment and sequencing. The raw data were filtered with QIIME2-dada2. The alpha diversity indexes including Chao1, Simpson, and Shannon index, and beta diversity were calculated with the “QIIME2” R package (v3.5.2). Pearson correlations between the metabolomics data and microbial taxa at the genus level were processed with corrplot R Package (version 4.0.5).

## Result

### Quality Control in Metabolomics

**Fig. 1:**
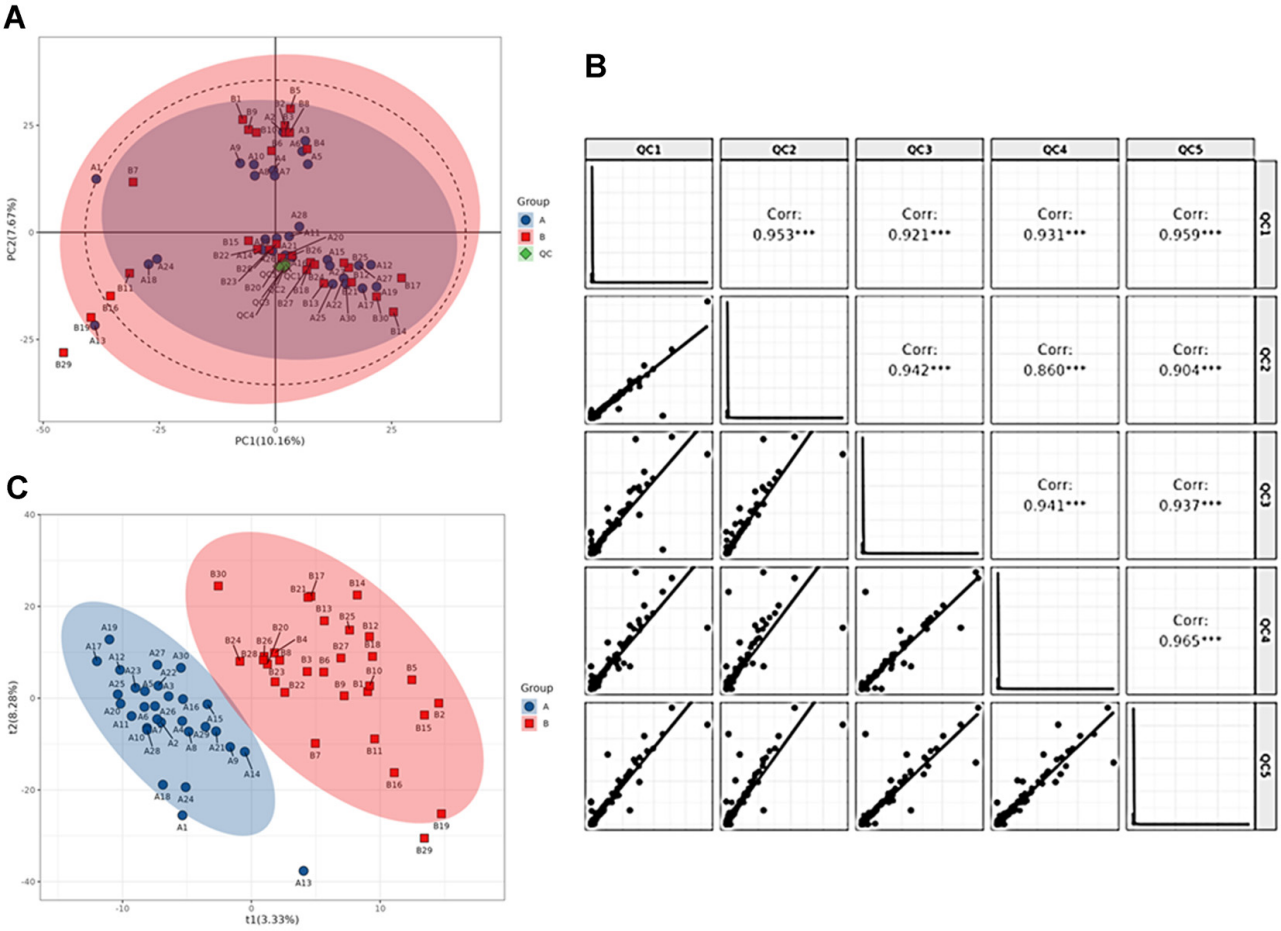
Quality Control (**QC**) in metabolomics. (**A**) The score chart of principal component analysis (**PCA**) analysis for the three samples under both positive and negative ion modes of metabolomics. QC: All quality control samples. A: The control group; B: the GDM group; (**B**) The diagram of the Correlation for five QC samples under both positive and negative ion modes. (**C**) The score plot of Orthogonal Partial Least Squares Discrimination Analysis (OPLS-DA) for the metabolites in Con and GDM groups.

The quality control (QC) of the metabolic datasets in this study was examined by principal component analysis (PCA). The ion peaks of metabolites were extracted using MSDIAL software, and a total of 50126 ion peaks were collected. The peaks extracted from all experimental and QC samples were subjected to UV PCA analysis with 7-fold cross-validation. In Fig. 1A, the score plots of principal component analysis (PCA) showed that the quality control (QC) samples clustered intensively and the correlation coefficients between QC samples were higher than 0.9 (Fig. 1B), validating that the quality control in this study was reliable.

### Identify Different Metabolites in GDM

**Fig. 2:**
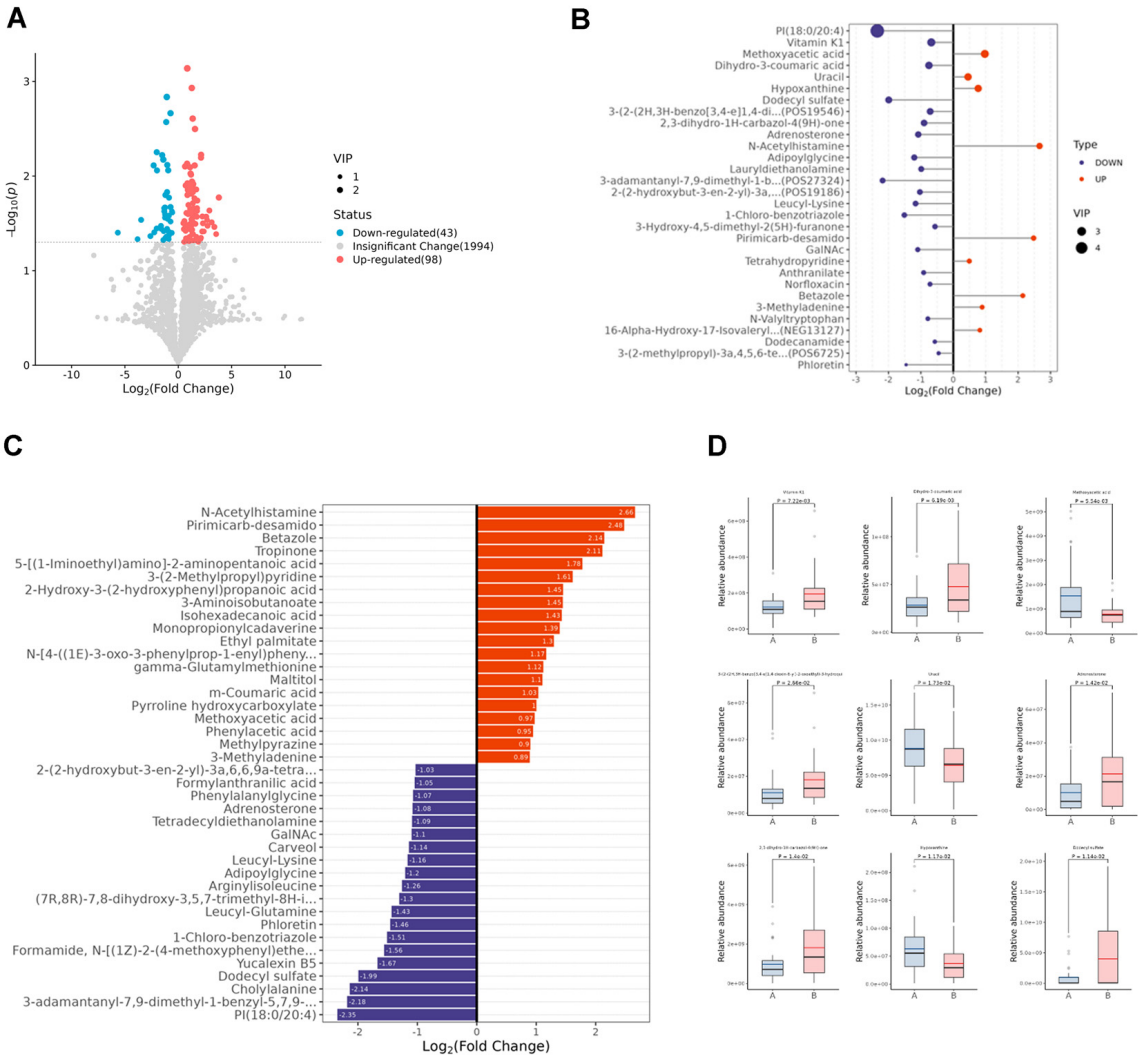
Identify different metabolites in Con and GDM groups. (**A**) Volcano Plot of the metabolites in Con vs. GDM. The differential expression genes were marked by different colors and shapes. (**B**) VIP plots of the top 30 differential metabolites in Con vs. GDM. The size of the dots corresponded to the value of OPLS-DA. (**C**) The bar chart of the top 40 differential metabolites in Con vs. GDM. (**D**) The relative abundance of top 9 differential metabolites in Con vs. GDM. P value was indicated.

As shown in Fig. 1A, the metabolites in the control (A) and GDM (B) groups clustered in a discriminable trend, indicating that there were some different metabolites between Con and GDM groups. To further screen the metabolites between the two groups, the collected datasets were processed for the subsequent multivariate analyses with a supervised orthogonal Partial Least Squares Discrimination Analysis (OPLS-DA), which can measure the influence strength and explanatory power of datasets based on VIP (Variable Importance for the Projection). Following the screening criteria [OPLS-DA VIP > 1, Fold Change (FC) >= 1.5 or =< 0.67 and T-test *p* < 0.05], Total 79 differential metabolites between the control (A) and PDM (B) groups were identified and the score chart of PLS-DA demonstrated a clear separated trend in OPLS-DA (Fig. 1C). Next, the datasets were processed for the univariate analysis with fold change (FC) analysis combined T-test and the screening criteria (Fold Change >=1.5 or =< 0.67 and/or T-test < 0.05) was set. The volcano plot indicated that there were 98 upregulated and 43 downregulated differential metabolites in Con vs. GDM (Fig. 2A). Depending on the above analysis, the top detailed 30 differential metabolites were listed in VIP plots (Fig. 2B) and the top detailed 40 differential metabolites were listed bar chart (Fig. 2C). The top 9 metabolites with the highest differential VIP in Con vs. GDM were selected and the relative abundance of all the 9 metabolites was significantly different with Welch’s T-test (Fig. 2D). The name of the metabolites was: Vitamin KI, Dihydro-3-coumaric acid, Methoxyacetic acid, 2-hydroxyquinoline, Uracil, Adrenosterone, Dihydro-carbazole, DodecyIsulfate, and Hypoxanthine.

### Enrichment Analysis in Differential Metabolites and Metabolic Pathways between Con and GDM Groups

**Fig. 3:**
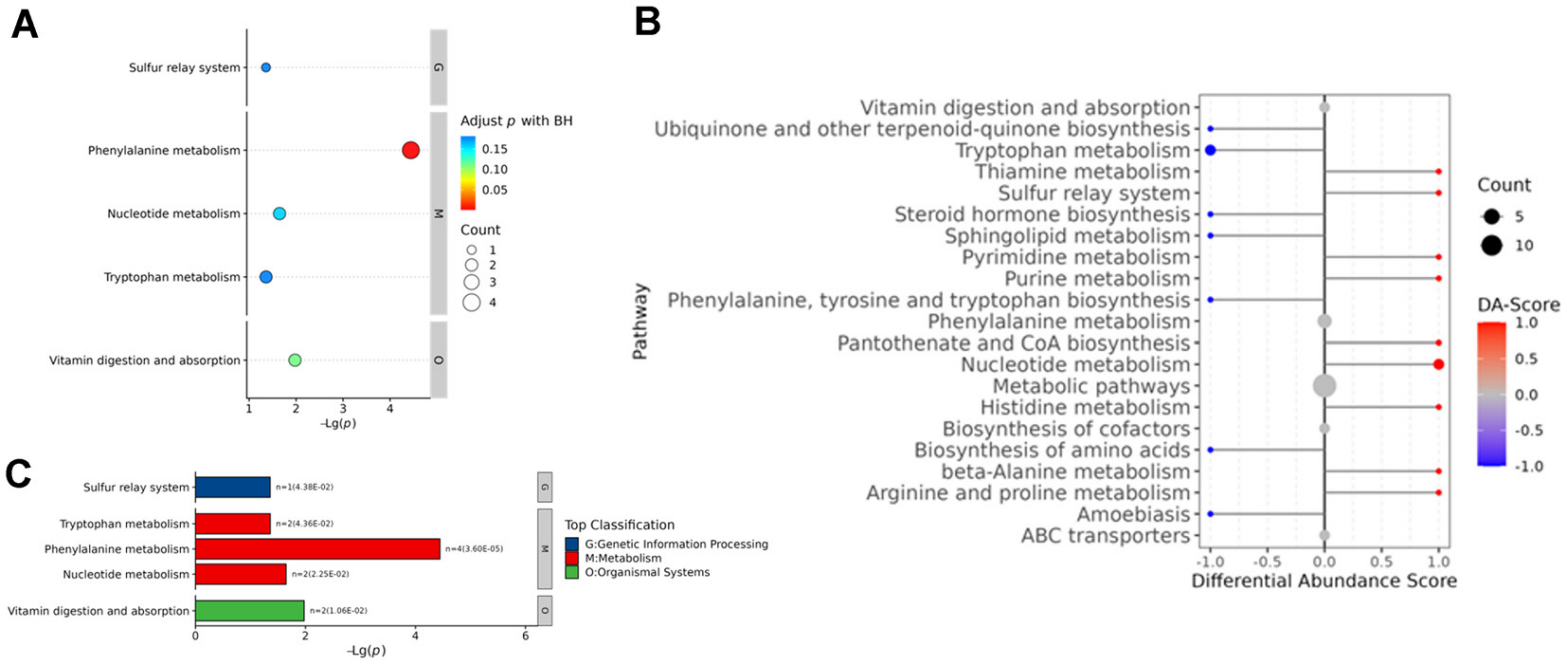
Enrichment analysis of differential metabolites. (**A, B**) KEGG pathway analysis for differential metabolites between the Con and GDM groups was presented with a Bubble diagram (**A**) and bar chart (**B**) at level 1 classification. (**C**) Differential abundance (DA) scores chart of differential metabolites between the Con and GDM.

To search the involved potential metabolic pathways among the altered metabolites between Con and GDM groups, the differential metabolites were classified with HMDB classification and processed for KEGG analysis. The top 2 differential metabolic pathways between Con and GDM groups were phenylalanine metabolism and Nucleotide metabolism following the KEGG level 1 classification (Fig. 3).

### Different Gut Microbial Communities between Con and GDM Groups

**Fig. 4:**
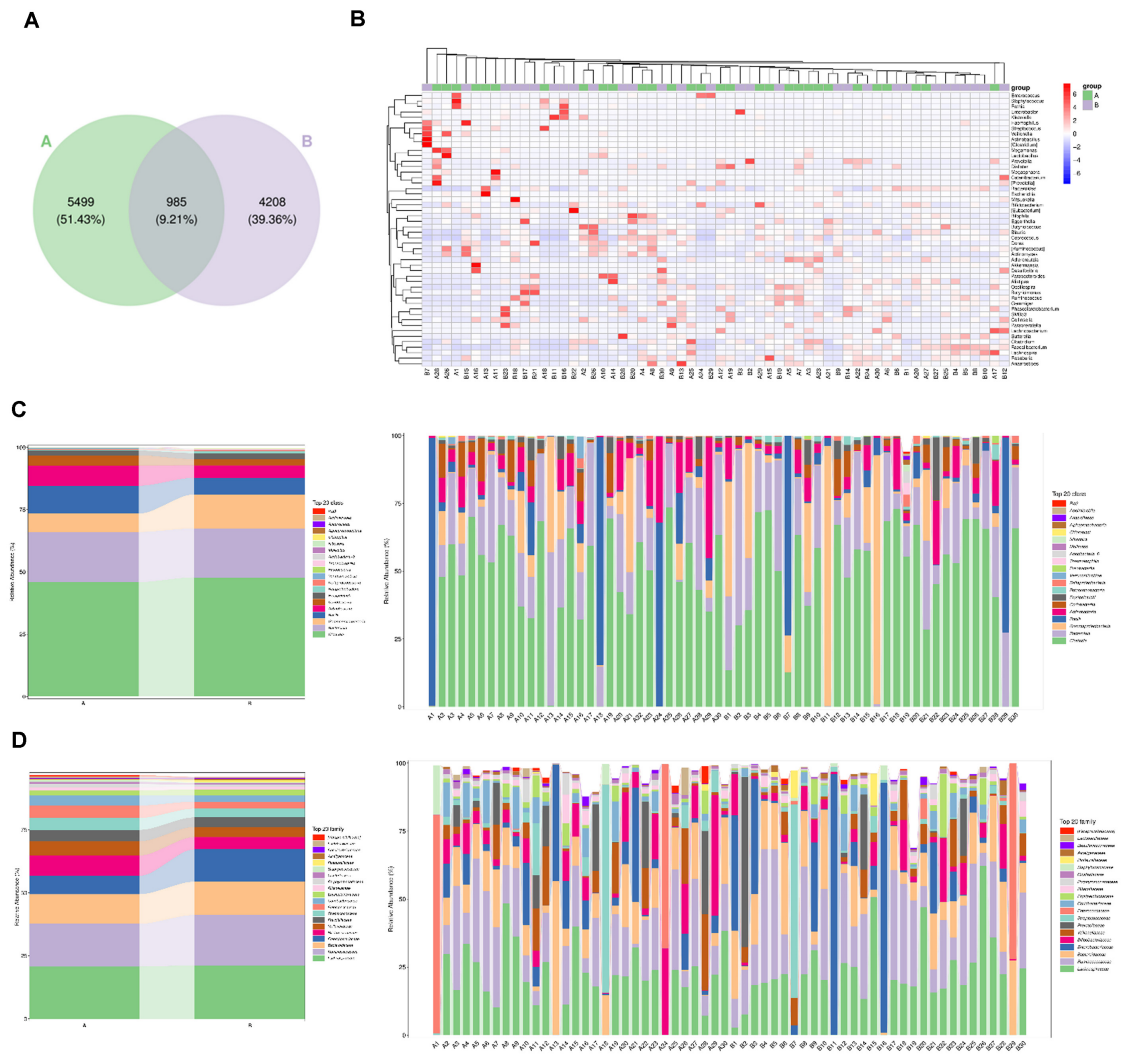
Comparison of gut microbial composition in Con and GDM groups. (**A**) Venn diagram of the operational taxonomic units (OTUs) identified in the microbe of the control and GDM groups. (**B**) The heatmap of the microbe composition at the species level between the control and GDM groups. (**C**) Top 20 abundance of microbial taxa at the class level in control and GDM groups. (**C**) Top 20 abundance of microbial taxa at the family level in control and GDM groups.

**Fig. 5:**
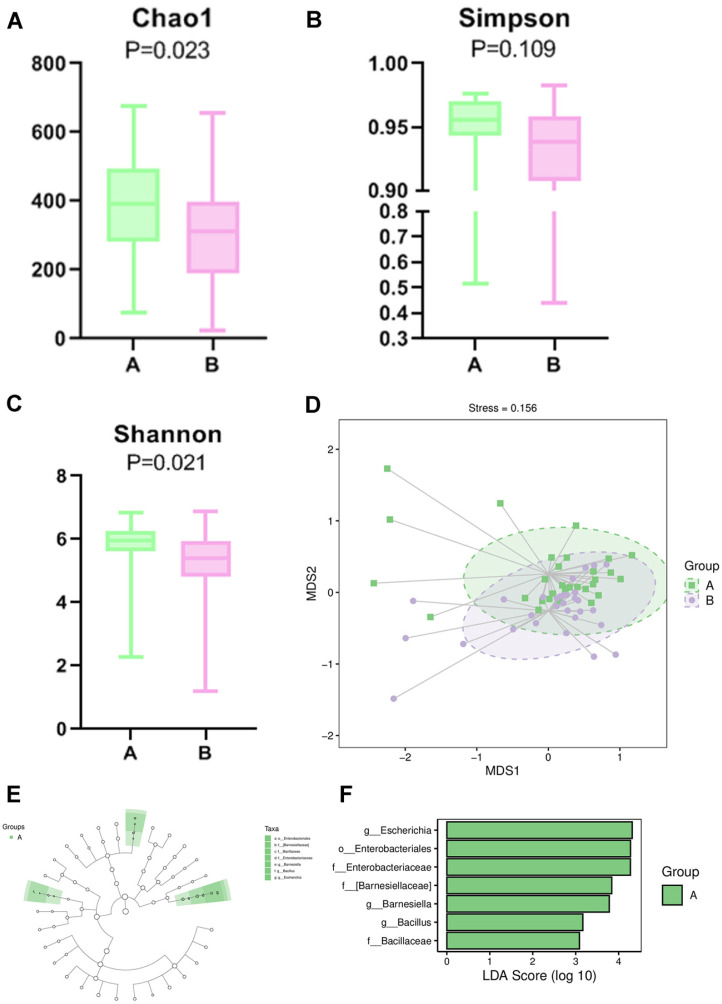
Diversity comparisons and LEFse analysis of gut microbiota between Con and GDM groups. (**A-C**) Microbial diversity of the control and GDM groups was estimated by three alpha diversity analyses: (**A**) Chao1 index, (**B**) Simpson index and (**C**) Shannon index. (**D**) beta diversity between the control and GDM groups was estimated by NMDS analyses. (**E, F**) LEfSe analysis of the control and GDM groups. (**E**) The cladogram of the gut microbiota in Con vs. GDM, q < 1X10E-5. (**F**) The significant biomarkers with LDA score ≥ 3 in Con vs. GDM groups.

The 16S rDNA sequencing data showed that there were 985 common OTUs between Con and GDM groups; 5,499 specific OTUs for Con and 4,208 for GDM groups, respectively. These group-specific OTUs indicated that there was a different gut microbiome of the hosts (Fig. 4A). We compared the gut microbial composition between control and GDM groups and found that there were significant differences at different Levels of microbe (Table 1 and Fig. 4B-4D). We estimated the gut microbial diversity between the Con and GDM groups via three alpha diversity metrics (Fig. 5A-5C). Gut microbial diversity was significantly lower in the GDM than in control with Chao1 (Fig. 5A) and Shannon Index analysis (Fig. 5B) but not different with Simpson Index (Fig. 5C). These indicated the decreasing trend of microbiota diversity (abundance and richness) in GDM group with alpha diversity analysis. NMDS analysis for beta diversity comparisons between Con and GDM groups showed that the Con and GDM groups were not clustered together and had differences (Fig. 5D). LEfSe (Linear discriminant analysis Effect Size) analysis was also performed to further investigate the microbiota with significant abundance differences in GDM. As shown in the cladogram of the gut microbiota in the GDP group (Fig. 5E) and the potential biomarkers with LDA values >3 (Fig. 5F). There were 3 main significant microbial genera (Escherichia, Barnesiella and Bacillaceae) in the GDM group.

### Potential Association of Metabolites to Microbiota between Con and GDM Groups

**Fig. 6:**
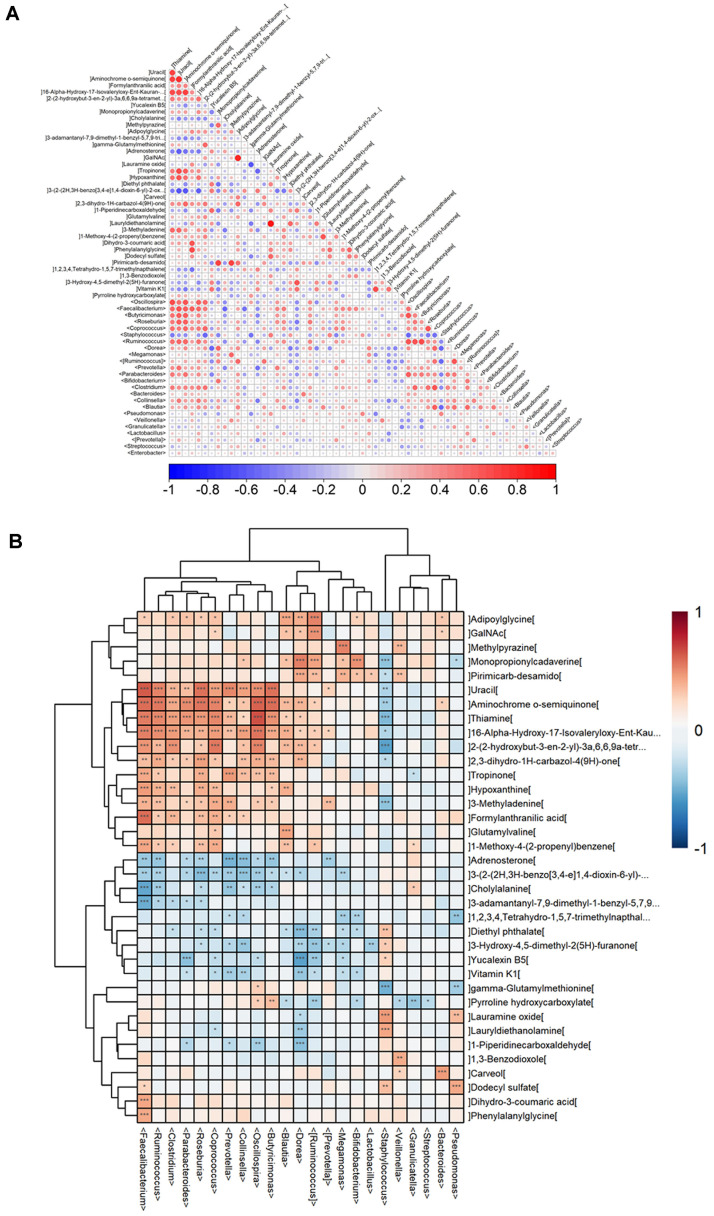
Correlation analysis for the differential metabolites and differential gut microbiota between Con and GDM groups. (**A**) The matrix heatmap of the correlation coefficient between the differential metabolites and differential gut microbiota in Con vs. GDM. (**B**) Hierarchical clustering heatmap of the correlation between the differential metabolites and differential gut microbiota n Con vs. GDM.

Altered gut microbiota may interact with fecal microbial metabolite profiles each other. The correlation analyses were performed to investigate the potential associations between the dysregulated metabolites and altered microbiota in the control and GDM groups. First Spearman correlation analysis was performed and the correlation coefficient was shown in the matrix heatmap. It indicated that metabolites were associated with microbiota (Fig. 6A). To reflect the correlation between metabolites and altered gut microbiota in more detail, Spearman correlation hierarchical clustering analysis was performed. Referring to the altered microbiota shown in Table 2, four genera: *Pseudomonas*, *Streptococcus*, *Staphylococcus*, and *Clostridium* had significant correlation to metabolites with hierarchical clustering analysis. *Pseudomonas*, *Streptococcus*, *Staphylococcus*, and *Clostridium* were shown to be significantly correlated with 5, 1, 17 and 12 metabolites, respectively. The top 2 genera were *Staphylococcus* and *Clostridium*. In the list of metabolites with significant correlation to microbiota with hierarchical clustering analysis, 13 metabolites belong to the top differential metabolites listed in Fig. 2B-2C. There were only four metabolites (Monopropionyl-cadaverine, 3-methyladenine, gamma-glutamylmethionine and Dodecyl sulfate) were associated with the four differential genera.

## Discussion

Gut microbial metabolites, including short-chain fatty acids, bile acids, and metabolites derived from amino acids, have been demonstrated to play a role in T2M and GDM. In this study, we examined fecal metabolomics, gut microbiome and their association in pregnant women with and without GDM. In fecal metabolomics, the top differential metabolites were Vitamin K, Dihydro-3-coumaric acid, Methoxyacetic acid,2-hydroxyquinoline, Uracil, Adrenosterone, dihydro -carbazolone, DodecyIsulfate and Hypoxanthine etc. the KEGG pathway analysis indicated the top 2 differential metabolic pathway between Con and GDM groups were phenylalanine metabolism and Nucleotide metabolism.

The data in this study showed that the increased Phenylalanylglycine, m-Coumaric acid and Phenylacetic acid among the top differential metabolites in GDM belonged to Phenylalanine. Phenylalanine (symbol Phe or F) [3] is an essential α-amino acid and a precursor for catecholamines (dopamine, norepinephrine, epinephrine) [13]. Meta-analysis demonstrated that both types of diabetes developed marked disturbances in amino acid metabolism with increased aromatic amino acids (AAA, phenylalanine, tyrosine, and tryptophan) and the phenylalanine, which might have an anti-incretin effect, decreasing cell uptake of glucose [14]. A recent study demonstrated that phenylalanine modified insulin receptor beta (IRβ), impaired insulin signaling and contributed to T2D pathogenesis. Phenylacetylglutamine (PAGln), a metabolite derived from microbial fermentation of phenylalanine to phenylacetic acid, was identified as a potential biomarker of T2M with distal symmetric polyneuropathy via metabolomics [15]. Targeted metabolomics revealed that the dysregulated phenylalanine metabolism including increased phenylalanine, phenylpyruvic acid and N-acetyl-L-phenylalanine were upregulated in AD brain tissues, indicating associated with AD pathology [16]. All these supported that the dysregulated of phenylalanine metabolism plays a critical role in GDM pathogenesis. There was a study identifying the circulating dopamine insufficiency as gut microbiota-driven metabolites linked to GDM progression [17]. Dopamine was derived from phenylalanine and regulated whole-body metabolism including glucose metabolism [18]. This suggested that the phenylalanine metabolism may be not only a mechanism underlying the gut microbiota linked to the development of GDM but also an event that serves as a promising target for therapeutic intervention.

Another notably altered metabolic pathway is nucleotide metabolism. A recent study indicated that Gut microbiota-mediated nucleotide synthesis attenuates the response to chemoradiotherapy in rectal cancer and uric acid is a potential prognosis marker for rectal cancer [19]. It also shows that gut Microbiota dysbiosis impacted purine metabolism [20] and pyrimidine metabolism which contributed to pathogenesis in APP/PS1 mice [21]. This supported that the dysregulated nucleotide metabolism might play a role in GDM. Uracil and hypoxanthine were top differential metabolites in GDM and involved in nucleotide metabolism. Studies indicated that uracil nucleotides (UDP and UTP) can activate purinergic signaling, which contributes to the pathogenesis of metabolic disorders including T2D [22]. Deamination of nucleobases forms xanthine (X), hypoxanthine (I), oxanine, and uracil, all of which are mutagenic and miscoding in DNA and interfere with transcription. Studies have revealed that dysregulation of purine metabolism including hypoxanthine was involved in diabetic complications [23]. These supported that uracil, hypoxanthine and nucleotide metabolism played a critical role in GDM pathogenesis.

For the gut microbiome, there were different alpha and beta diversity between control and GDM. The different relative abundance at the class level was c_unclassified_Firmicutes between the control and GDM groups. There were 7 differential bacterial OTUs at the genus level, including primarily *Allobaculum*, *unclassed Firmicutes*, *Clostridiacea*, *Pseudomonas* and *Lachnospiraceae*, etc. This was consistent with other studies, demonstrating that there was a significant increase in the relative abundance of Firmicutes in the GDM group [24]. A previous study reported that *Lachnospiraceae* OTUs, belonging to *c_Firmicutes*, were positively associated with type 2 diabetes mellitus (T2D) [25].

To further explore the role of gut microbiota and its related (fecal) metabolites, the potential association of metabolites to microbiota was analyzed. We focus on the association between the top differential metabolites and the altered gut microbiota. It showed that the two strongest gut microbiotas at the family level associated with altered metabolites were Staphylococcus and *Clostridium*, both of which belong to *c_Firmicutes*. Notedly, *Clostridium* was increased in GDM and positively associated with the metabolites (*i.e.*, Uracil, Aminochrome o-semiquinone and Thiamine) while Staphylococcus was negatively associated with the metabolites (*i.e.*, Uracil, Aminochrome o-semiquinone and Thiamine). These indicated that both Staphylococcus and *Clostridium* were involved in GDM via altered metabolites in different ways. A study showed that the gut symbiont *Clostridium* sporogenes generated phenylalanine acid metabolites through metabolizing phenylalanine to phenylpropionic acid (PPA) or phenylacetic acid (PAA). A recent study showed that PAA served as the precursor of the gut microbiota-generated metabolite phenylacetylglutamine (PAGln) and phenylacetylglycine (PAGly), which promoted the pathogenesis of diseases [26, 27]. In summary, the significant differential gut symbiont *Clostridium* was linked to the dysregulation of phenylalanine metabolism in GDM. Several clinical trials demonstrated that certain probiotics, *i.e.* *Lactobacillus* and *Bifidobacterium* species, can ameliorate the insulin sensitivity glucose control in T2D patients. The fecal microbiota transplantation has been successfully applied in the treatment of *Clostridioides* difficile infection and is a potential therapy for T2DM.

## Conclusion

In this study, we investigated the gut microbial, and fecal metabolites and their association in gestational diabetes mellitus. The metabolome indicated that the top 2 differential metabolic pathways between Con and GDM groups were phenylalanine metabolism and Nucleotide metabolism. The proficiently altered gut microbiota at the class level was *c_unclassified_Firmicutes*. Correlation analysis between microbiota and metabolites showed that the increased gut symbiont *Clostridium* was linked to the dysregulation of phenylalanine metabolism in GDM. These findings may provide a unified mechanism to explain how microbe–metabolite association contributes to GDM pathogenesis and also be a novel therapeutic strategy to treat GDM.

## Figures and Tables

**Table 1:** The general information of patients.

	Control	GDM	p
Age	27.38 ± 4.45	29.4 ± 3.74	0.064
BMI	25.09 ± 3.22	27.29 ± 3.47	0.013
Gestational age during examination	38.62 ± 1.37	38.67 ± 1.35	0.897
Delivery age	41.13 ± 13.07	39 ± 1.17	0.377
Systolic blood pressure (mmHg)	112 ± 19.30	116.07 ± 6.47	0.278
Diastolic blood pressure (mmHg)	72.21 ± 4.83	71.07 ± 5.04	0.379
Weight gain (kg)	10.10 ± 4.39	13.27 ± 5.03	0.013
Family history of diabetes	NA	NA	/
Medical history	NA	NA	/
Glucose at 0 min (mmol/l)	4.54 ± 0.29	4.81 ± 0.47	0.009
Blood glucose at 60 min (mmol/l)	7.74 ± 1.28	10.30 ± 1.88	<0.001
Blood glucose at 120 min (mmol/l)	6.42 ± 1.11	8.76 ± 1.40	<0.001

**Table 2:** The differential gut microbiota between Con and GDM groups.

Level	Name
Phylum	p__Actinobacteria (p=0.049)
Class	c_unclassified_Firmicutes
Order	o__Pseudomonadales (p=0.006); o__unclassified_Firmicutes (p=0.022)
Family	f__Bacillaceae (p=0.007); f__Pseudomonadace (p=0.030) f__Carnobacteriaceae (p=0.024); f__unclassified_Firmicutes (p=0.022); f__Planococcaceae (p=0.037)
Genus	g__unclassified_Lachnospiraceae (p=0.035); g__Pseudomonas (p=0.030) g__Granulicatella (p=0.047); g__Barnesiella (p=0.021) g__Allobaculum (p=0.005); g__unclassified_Firmicutes (p=0.022 g__unclassified_Clostridiaceae (p=0.024)
